# Prevalence and correlates of subjective cognitive concerns in Australian university students during the COVID-19 pandemic

**DOI:** 10.3389/fpsyg.2022.1094497

**Published:** 2023-01-11

**Authors:** Laura J. Bird, Melinda McCabe, Yen Ying Lim, Kim Cornish

**Affiliations:** Turner Institute for Brain and Mental Health, School of Psychological Sciences, Monash University, Clayton, Melbourne, VIC, Australia

**Keywords:** cognition, subjective cognitive concerns, COVID-19, pandemic, university students

## Abstract

**Introduction:**

Coronavirus (COVID-19) instigated unprecedented global effects on healthcare systems, economies, employment, education, travel, and social lives. In addition to increased mental health challenges, pandemic restrictions have triggered emerging cognitive concerns. University students are at particularly high risk of adverse lockdown-related effects, yet despite the substantial adaptions to learning necessitated by COVID-19, limited research has so far focused on the cognitive consequences of the pandemic among university students. This study aimed to comprehensively examine the nature, prevalence, and correlates of subjective cognitive concerns among 972 students (Median age = 22 years, 70% female) enrolled at Monash University, Australia, in December 2020.

**Methods:**

Students completed the online THRIVE@Monash survey, 5 weeks following prolonged lockdown in Melbourne. Using group comparisons and hierarchical binary logistic regression analyses, we examined associations between demographic and enrolment characteristics, COVID-19-related experiences and impacts (author-developed questions), self-reported anxiety and depression symptoms (PROMIS Anxiety and Depression scales), and students’ perceived changes in everyday cognitive functions (author-developed questions).

**Results:**

Over 60% of students reported subjective cognitive concerns (SCCs). After controlling for anxiety and depression symptoms, students reporting more SCCs were more likely to be younger, from White/European ethnic backgrounds, and in their first year of undergraduate study. No differences in SCCs were found between male and female students. Greater worry, anxiety, or stress related to COVID-19 (e.g., infection, leaving the house, hygiene and exposure prevention, impact on physical and mental health), and time spent reading or talking about COVID-19, were generally not associated with SCCs after controlling for anxiety and depression symptoms.

**Discussion:**

These findings highlight vulnerable subgroups of students who might benefit from regular monitoring, education, and interventions to support their cognitive health during the pandemic and beyond. In addition, cognitive concerns may provide additional insight into mental health problems among students, and emphasize the importance of understanding factors that impact students’ long-term academic and career success.

## 1. Introduction

The coronavirus disease (COVID-19) outbreak was declared a global pandemic by the World Health Organization in March 2020 (World Health Organization, 2020a). By the end of December, over 79 million confirmed cases and more than 1.7 million total deaths were reported globally (World Health Organization, 2020b). In Australia, while case numbers relative to population were significantly lower compared to the United States, United Kingdom, and other upper-middle income countries (Dong et al., 2020; Johns Hopkins University, 2020), lockdown restrictions were some of the harshest. Melbourne is now renowned for enduring the most cumulative lockdown days (267 days) of any city in the world (Kelly, 2021).

COVID-19 control measures have affected mental health and psychological wellbeing across countries and subpopulations (Gloster et al., 2020; Khan et al., 2020). For example, in Italy, high rates of posttraumatic stress, anxiety, depression, insomnia and other symptoms were reported across March and April 2020 (Rossi et al., 2020), even after the effects of pre-existing psychological trauma or psychiatric diagnoses were controlled for. These symptoms were attributed to lockdown restrictions and associated with adverse COVID-19-related experiences such as loss of work or increased workload, being quarantined, or having a loved one diagnosed with or die from COVID-19. In addition, younger age and being female were associated with more severe mental health symptoms. Relatively less research has focused on the impact of COVID-19 restrictions on cognitive function, although similar patterns are emerging. Specifically, factors such as lockdown confinement and changes to employment, self-reported vulnerability to stress (e.g., emotional suppression, lack of social support), poor general health, younger age and being female were associated with poorer mood and more physical symptoms (e.g., pain, fatigue, sleepiness), which were in turn associated with significantly greater subjective cognitive concerns (SCC) (Fiorenzato et al., 2021; Podlesek et al., 2021; Torrente et al., 2022).

Cognitive concerns are pertinent in tertiary educational settings, even outside of a pandemic context, and particularly in students with concurrent psychiatric symptoms (Glober and Suhr, 2020; Karr and White, 2021). Prolonged lockdown restrictions in 2020 necessitated a transition to remote online learning, a novel and challenging experience for many students and educators alike (Casacchia et al., 2021). Together with an increase in COVID-19-related mental health issues including depression and anxiety symptoms, and loneliness (Cao et al., 2020; Xiong et al., 2020; Arslan and Coşkun, 2022), university students represent a population at risk for cognitive disturbances. Concerns such as difficulties in the ability to concentrate and focus thoughts, learn and recall relevant and new information, may have a prolonged adverse impact on students as they complete their degrees and enter the workforce over the coming years. It is therefore critical that tertiary education providers monitor cognitive concerns reported by students, and provide appropriate and effective support and interventions for those at most risk.

In a 2020 survey of Australian university students, Liu et al. (2021) identified White/European ethnicity, stress relating to restrictions, mental health-related worry, worsened diet, perceived insufficiency of physical distancing communication methods (e.g., phone/video call, social media, email), and social isolation as negative predictors of psychological wellbeing. Given the relationship between cognitive concerns and psychological symptomatology (Giusti et al., 2020; Podlesek et al., 2021), similar factors may predict SCCs among university students. To date, two main studies have specifically explored the cognitive consequences of COVID-19 restrictions within tertiary students, with both observing high levels of subjective difficulties in concentration and learning abilities due to pandemic-related distance education (Giusti et al., 2021; Pisano et al., 2021). In one study, both self-reported memory difficulties and objectively-measured working memory and prospective memory performance were worse 1 month following COVID-19 confinement (Pisano et al., 2021).

This cross-sectional study aims to determine the nature of SCCs in tertiary education students enrolled at the largest Australian university (Monash University; Australian Government, 2022), and examine demographic and COVID-19-related correlates of cognitive concerns. Consistent with the extant literature, the first hypothesis was that younger age, self-identified female gender, and White-European ethnic background will be associated with greater SCCs. The second hypothesis expected greater SCCs to be associated with increased stress around COVID-19-related restrictions, greater worry/anxiety about and consumption of mass media regarding the pandemic. Finally, given the unique challenges faced by first-year university students (Maymon and Hall, 2021), this study explored the influence of degree type (undergraduate vs. postgraduate) and year level on SCCs.

## 2. Materials and methods

### 2.1. Participants

Students at Monash University (living both on and off campus) were invited to participate in THRIVE@Monash, a series of (ongoing) online surveys capturing data at 5 timepoints throughout 2020. A convenience sample of 972 participants completed the THRIVE@Monash survey in December 2020, 5 weeks after a 112-day community-wide lockdown in Melbourne, Australia. This sample size is sufficient to capture small effect sizes for comparisons of SCC endorsement across participant subgroups, concordant with recent studies (e.g., *d* values ranging from as low as |0.02| to more than |2.00|, η_p_^2^ from |0.01| to |0.15|) (Fiorenzato et al., 2021; Pisano et al., 2021; Podlesek et al., 2021). The lockdown period was characterized by restricted local travel radius (no greater than 5 km from home) and “stay at home” orders, curfews, closing of retail and other businesses, online learning for school-aged and tertiary students, and closing of interstate and international borders. The study was approved by Monash University Human Research Ethics Committee (Project ID: 23969).

### 2.2. Measures

Where available, measures in this study comprised existing, well-validated instruments that are used among clinical and non-clinical populations (e.g., PROMIS Anxiety and Depression scales, see below). Other survey measures were developed by the authors, given the rapid response required to the emerging pandemic situation in March 2020 in Australia, and the limited extant research on the effects of COVID-19 at the time. Development of these survey questions (e.g., subjective cognitive concerns, COVID-19-related items) was guided as much as possible by the COVID-19 research emerging from China, United States, and United Kingdom at the time, and in consultation with experts within the research team’s network. All items were determined to have face validity by academic and student researchers within the team prior to survey launch.

#### 2.2.1. Demographics

Demographic information extracted for the current study included age (years), gender identity (female, male, non-binary/gender diverse, gender not listed, or prefer not to say), ethnicity (Aboriginal and/or Torres Strait Islander, South East Asian, East Asian, South Asian, White/European, African, or Other), year of study (first, second, third, or fourth year and higher of their current degree), and status as an undergraduate or postgraduate student.

#### 2.2.2. Subjective cognitive concerns

Six items were developed and adapted for young adults by the authors based on established scales of subjective cognitive concerns in older adults (e.g., MAC-Q; Crook et al., 1992; Cognitive Function Instrument; Walsh et al., 2006), to assess the presence and severity of perceived changes in cognitive abilities across four domains (confidence in learning, concentration, muddled thoughts, memory recall; see Supplementary Material for further details).

#### 2.2.3. COVID-19 exposure, worry, anxiety, and behavior

Five groups of questions designed by the authors explored aspects of COVID-19-related exposure (i.e., diagnostic status), worry (e.g., about infection, physical or mental health impact, staying safe), stress surrounding restrictions on leaving the home, anxiety regarding exposure prevention and hygiene, and behaviour (time spent reading or talking about COVID-19) over the prior 2 weeks (see Supplementary Material).

#### 2.2.4. Anxiety and depression

Self-reported symptoms over the prior 7 days were assessed by the Patient-Reported Outcomes Measurement Information System (PROMIS) Anxiety and Depression scales (Choi et al., 2014; Schalet et al., 2014), with higher total scores reflecting greater symptom levels (see Supplementary Material).

### 2.3. Procedure

Data were collected between December 9–15, 2020, through an anonymous survey link *via* the Monash secure Qualtrics service. An invitation and short description of the survey was sent *via* email to all students enrolled in an Australian campus of Monash University. In order to obtain a representative sample of students, the research team liaised with student groups around Australian campuses of Monash University and the University Marketing and Communications team, who directly emailed and posted promotions of the survey across multiple student groups. As the THRIVE@Monash survey series began in May 2020, the December data collection timepoint represents the fifth opportunity for students to engage in these surveys across 2020. Students indicated their consent to participate at the beginning of the survey and confirmed their eligibility for the study. Only participants who were 18 years or older, enrolled at Monash at the time of survey, and were able to provide informed consent to participate were included. A subsample of the study (those living on campus) were offered an opportunity to win a $50 digital gift card upon completion of a larger version of the survey. The rest of the sample was not offered any reimbursement. The survey was open to students for completion over approximately 4 days (opening 9:00 am on the first day and closing at 11:59 pm on the last day). The limited timeframe for completion was used to control for the frequent changes in COVID-19 restrictions.

### 2.4. Statistical analyses

Data were analysed using SPSS version 27.0.1.0. We examined overall presence of SCCs on at least one out of four domains, with post-hoc comparisons for individual cognitive domains to better characterize specific SCCs endorsed by students. Descriptive statistics were calculated for cognitive variables and the demographic and COVID-19-related correlates. Where continuous and scale variables of interest are non-normally distributed (indicated by significant Kolmogorov–Smirnov tests, data not shown), data were expressed as median (Q1, Q3). Number and percentage were reported for categorical variables.

To test the first set of hypotheses predicting greater SCCs in younger, female, and White-European students, Mann–Whitney U non-parametric comparisons of age between students endorsing versus not endorsing SCCs were conducted. South Asian and South East Asian ethnicity groups were combined into one category, and African was included in the “Other” category. This was due to small sample sizes in these subgroups. This resulted in four dummy-coded subgroups: East Asian, White/European, South/South East Asian, and Other. Fisher’s exact tests examined SCC frequencies between males and females, and for each ethnicity group. Hierarchical binary logistic regression analyses were conducted to examine associations between age, gender, and ethnicity (in separate models), and SCC endorsement. Self-rated anxiety and depression scores were included as covariates. For age, these analyses were repeated within age subgroups of 18–25 years and 26–45 years, in accordance with Fiorenzato’s et al. (2021) findings of lockdown-related worsening of cognition exclusively in 18–25-year-olds and 26–45-year-olds. Notably, only *n* = 33 students in the present study were aged >45 years. Outliers detected through the logistic regression analyses (cases with studentized residuals >2) were checked for their influence on the models, and odds ratios (OR) with 95% confidence intervals (CI) are reported.

To test the second set of hypotheses, associations between SCCs and COVID-19-related worry and exposure anxiety were explored with a series of Mann–Whitney U tests comparing composite worry/anxiety scores between students endorsing versus not endorsing SCCs. The COVID-19-related worry composite and exposure prevention and hygiene anxiety composite scores were subjected to a Box-Cox (power) transformation to reduce the significant skewness for these variables (Osborne, 2010). Fisher’s exact tests compared SCC endorsement between students reporting low vs. high stress regarding restrictions on leaving the home, and low vs. high amounts of time spent reading or talking about COVID-19. Logistic regression models examined the influence of restrictions-related stress and time spent reading and talking about COVID-19 on SCC endorsement, after controlling for anxiety and depression scores.

To explore associations between cognitive concerns and degree type and year, Fisher’s exact tests and Pearson Chi-squared test, respectively, compared the frequency of SCCs endorsed by undergraduate versus postgraduate students and across university year levels. Logistic regression models examined the influence of degree type and year level on SCC endorsement, after controlling for anxiety and depression scores.

Data were systematically missing across some demographic variables (including age, gender, ethnic background, and degree-related variables) due to an oversight in initial design of the online survey which allowed participants to skip past some questions (this was later corrected). In addition, not all students decided to respond to the questions regarding SCCs. Minimal data (2–3%) were missing for COVID-19-related variables. Given the amount and pattern of missing data across variables of interest, it was deemed inappropriate to perform missing data imputations. Sample sizes across analyses therefore differ, and results were interpreted with necessary caution where sample sizes were lower (see Supplementary Figure S1 for participant flowchart).

Statistical significance was indicated by two-tailed α = 0.05 for all analyses. Given the paucity of research specifically investigating the correlates of SCCs relating to COVID-19 in university students, analyses were not subjected to correction for multiple comparisons as we wished to explore all relevant emerging patterns from the data.

## 3. Results

### 3.1. Demographic characteristics

Table 1 displays the demographic characteristics of the entire participant sample with valid data relating to SCCs (*N* = 901, see Supplementary Figure S1). They largely comprised full-time young university students (although with an age range of 18–79 years), with a higher proportion of females.

**Table 1:** Demographic characteristics of the student sample (*N* = 901).

	Students with available data (*n*)	Static
		**Med (Q1, Q3)**
Age (years)		657	22 (20, 26)
		***n* (%)**
Gender		659	
Female			467 (70.9)
Male			171 (25.9)
Non-binary/gender diverse			12 (1.8)
Other			9 (1.4)
Ethnic background^a^		640	
East Asian			437 (68.3)
White/European			127 (19.8)
South Asian			38 (5.9)
South East Asian			34 (5.3)
African			3 (0.5)
Australian Indigenous/Torres Strait Islander			0 (0)
Other			19 (3.0)
Degree type^b^		355	
Undergraduate			188 (53.0)
Postgraduate			165 (46.5)
Year of degree		355	
First			157 (44.2)
Second			110 (31.0)
Third			59 (16.6)
Fourth or higher			29 (8.2)

^a^Some students endorsed multiple ethnic backgrounds, thus the total *n* across subgroups is > 640; ^b^*n* = 2 students reported their degree type as ‘other’.

### 3.2. Prevalence of subjective cognitive concerns

**Figure 1:**
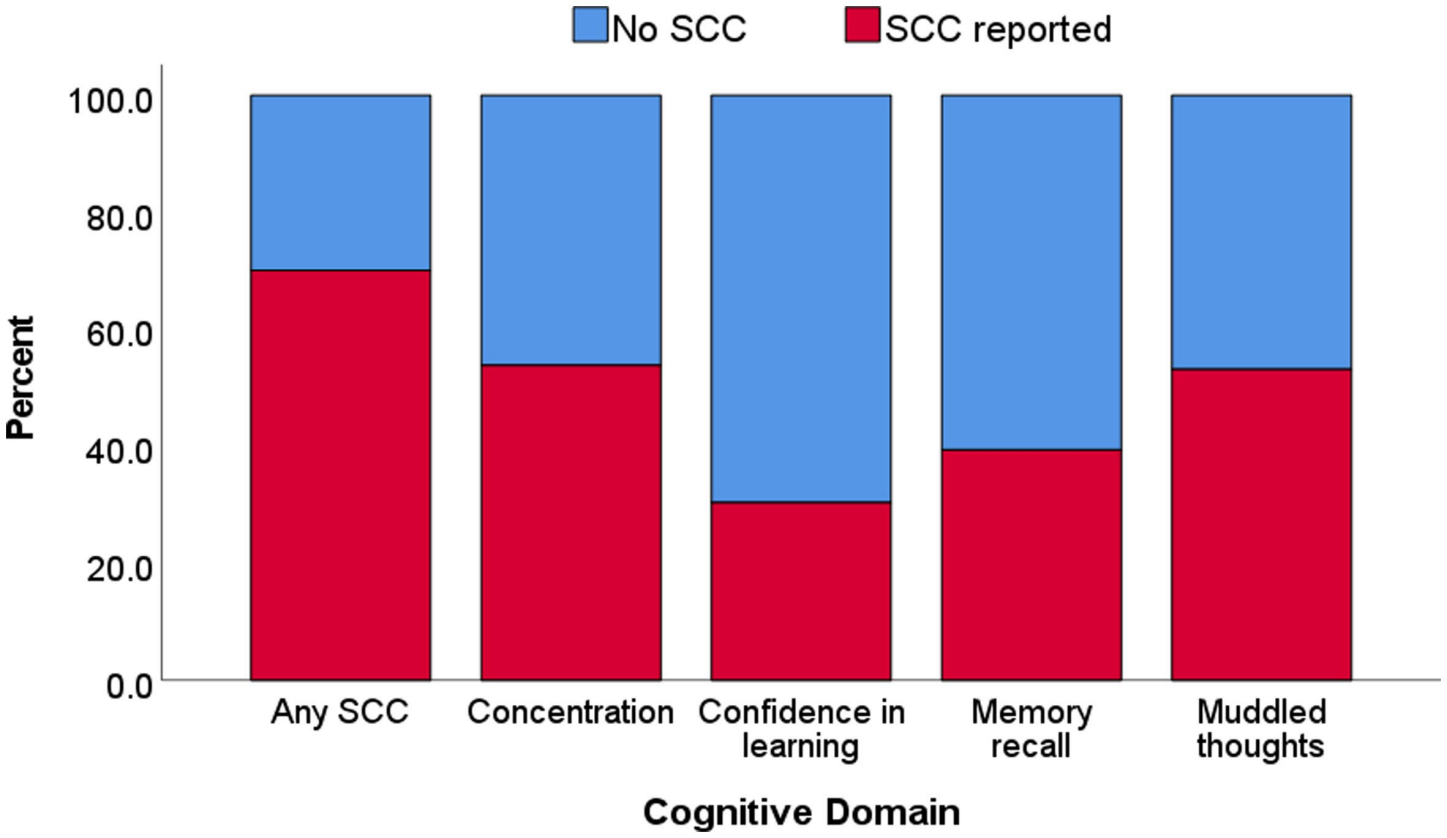
Percentages of students (scaled to 100%) endorsing subjective cognitive concerns (SCCs) relating to any domain (*n* = 901), concentration (*n* = 895), confidence in learning (*n* = 895), memory recall (*n* = 895), and muddled thoughts (*n* = 897). Percentages based on number of students with valid survey responses for each domain (i.e., excluding missing values).

Of the 972 students who completed the December 2020 THRIVE@Monash survey timepoint, 901 students responded to one or more of the questions about cognitive concerns. Overall, 63% (*n* = 564) endorsed concerns in at least one of the four cognitive domains (Figure 1). Increased difficulties in concentrating (46%) and experiencing muddled thoughts (45%) were the most frequently endorsed, followed by memory recall (32%) and confidence in learning (24%). Students located in the state of Victoria (66%), where 2020 lockdown restrictions were the most severe, more frequently reported SCCs overall compared to those studying from elsewhere in Australia (56%). This difference did not reach statistical significance, however, sample sizes were uneven with a small number of students studying outside (*n* = 50) compared to within Victoria (*n* = 718). There was a trend toward more Victorian students endorsing specific concerns about concentration (50% vs. 36%, *p* = 0.058).

Overall, 38% of students reported general concern about their memory or thinking abilities, and 23% (of *n* = 343 with valid responses) indicated they had spoken to someone about their cognitive concerns. Among students who endorsed no adverse changes in cognition, 23% still reported some overall concern about memory or thinking abilities (vs. 48% of students endorsing any SCCs, *p* < 0.001), and paradoxically, 36% reported they had spoken to someone about these concerns (vs. 20% of students endorsing any SCCs, *p* = 0.003). Across the whole cohort, students with higher anxiety were 5% more likely to endorse SCCs (*p* < 0.001).

### 3.3. Demographic characteristics of students endorsing SCCs

#### 3.3.1. Age

Students endorsing SCCs on at least one domain were significantly younger [22.0 (20.0, 25.0) years] than those reporting no SCCs [23.0 (20.0, 26.0) years; *U* = 43316.5, *p* = 0.002]. Beyond the effects of anxiety and depression, older age contributed to a reduced likelihood of endorsing SCCs, but only among students aged 18–25 years (OR = 0.86, 95% CI = 0.78–0.95, *p* = 0.002). This effect was observed specifically for the domains of concentration (OR = 0.91, 95% CI = 0.83–0.99, *p* = 0.035) and muddled thoughts (OR = 0.80, 95% CI = 0.72–0.88, *p* < 0.001).

#### 3.3.2. Gender

Less than 2% of students identified as non-binary/gender diverse. Thus, the following analyses focused on differences between male- and female-identifying students who responded to survey questions about SCCs. Males and females did not differ in the frequency of SCCs reported across all domains, except concentration. Females were more likely to endorse concerns about concentration difficulties than males (48% vs. 39%, respectively, *p* = 0.039; see Table 2). However, after controlling for anxiety and depression symptoms, there was no significant effect of gender on the likelihood of endorsing SCCs overall, or for any of the four domains. Accordingly, being female was associated with higher anxiety scores (Pearson *r* = −0.11, *p* = 0.005), but not depression.

**Table 2:** Frequency (*n*, %) of endorsed SCCs across cognitive domains within participant subgroups.

	Cognitive domain					Any SCC	Confidence in learning	Concentration	Muddled thoughts	Memory recall
Gender						
Female		297 (63.6)	114 (24.5)	223 (48.1)^^^	217 (46.7)	155 (33.5)
Male		103 (60.2)	44 (25.9)	66 (38.8)^^^	76 (44.4)	48 (28.1)
Ethnic background						
East Asian		255 (58.4)^**^	99 (22.8)	183 (42.2)^**^	180 (41.4)^***^	124 (28.6)^**^
White/European		95 (74.8)^***^	30 (23.6)	68 (53.5)^†^	73 (57.9)^**^	58 (46.0)^***^
South/South East Asian		48 (67.6)	24 (33.8)^†^	37 (52.1)	37 (52.9)	19 (27.1)
Other		15 (68.2)	10 (45.5)^^^	13 (61.9)	12 (54.5)	8 (36.4)
Degree type						
Undergraduate		126 (67.0)^**^	56 (29.9)	88 (47.3)^**^	94 (50.5)^***^	62 (33.3)
Postgraduate		86 (52.1)^**^	43 (26.4)	53 (32.5)^**^	46 (28.0)^***^	40 (24.5)
Year level						
First		87 (55.4)	41 (26.5)	55 (35.3)	54 (34.4)	41 (26.3)
Second		69 (62.7)	36 (33.0)	46 (42.2)	46 (42.2)	30 (27.5)
Third		37 (62.7)	18 (30.5)	28 (48.3)	28 (47.5)	25 (42.4)
Fourth or higher		19 (65.5)	4 (13.8)	12 (42.9)	12 (44.4)	6 (22.2)

Statistical significance indicators refer to Fisher’s exact tests, not including the covarying effects of anxiety and depression scores.

^**^p ≤ 0.01; ^***^p ≤ 0.001; ^†^*p* = 0.058; ^^^*p* = 0.039.

#### 3.3.3. Ethnic background

Frequencies of endorsed SCCs across subgroups are displayed in Table 2. Generally, East Asian students had less frequent concerns, while White/European students reported more frequent concerns, across all domains except confidence in learning. Students endorsing other and South/South East Asian ethnic backgrounds were more likely to endorse reduced confidence in learning. After controlling for anxiety and depression, the likelihood of endorsing SCCs overall remained higher for White/European students (OR = 1.84, 95% CI = 1.14–2.95, *p* = 0.012), and was specifically attributable to greater endorsement of muddled thoughts (OR = 1.78, 95% CI = 1.16–2.74, *p* = 0.009) and memory recall concerns (OR = 2.11, 95% CI = 1.35–3.28, *p* < 0.001). The lower likelihood of endorsing SCCs among East Asian students trended toward significance (OR = 0.68, 95% CI = 0.46–1.02, *p* = 0.061). The remaining ethnic groups did not significantly contribute to SCC endorsement beyond the effects of anxiety and depression.

### 3.4. Influence of COVID-19-related experiences on SCCs

#### 3.4.1. COVID-19 exposure and worry

Most students (*n* = 846, 94%) reported not knowing anyone within or outside their household who had been diagnosed with COVID-19 over the prior 2 weeks. Endorsement of SCCs was not significantly different in the minority of students who reported a COVID-19 diagnosis in either a member of the household (*n* = 8) or a non-member of the household (*n* = 36).

**Figure 2:**
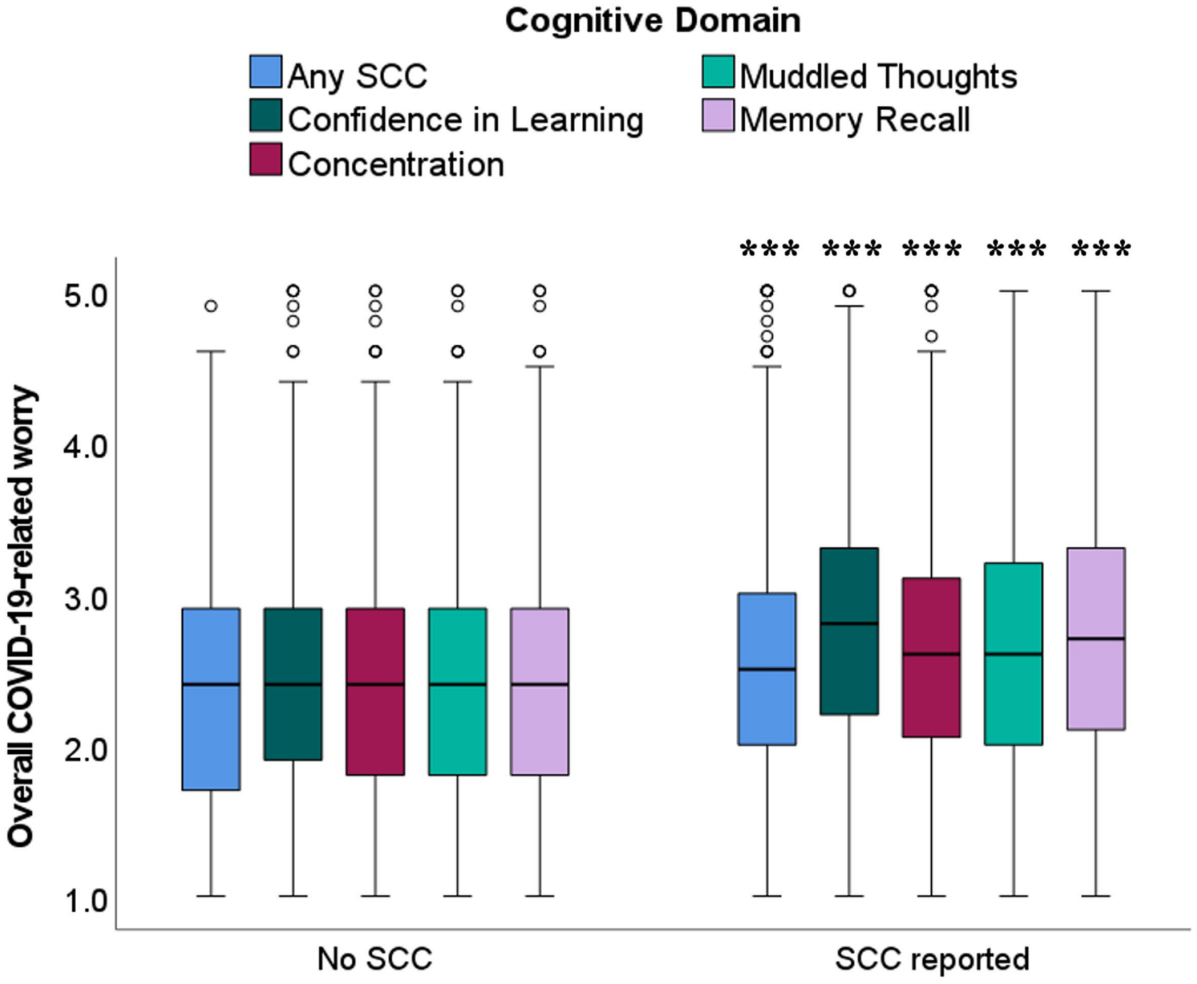
Boxplots illustrating higher median scores for COVID-19-related worry in students reporting the presence of SCCs, across all cognitive domains. ***p < 0.001, Mann–Whitney *U* comparisons between students endorsing presence vs. absence of SCCs within each cognitive domain and overall. Not adjusted for differences in self-reported anxiety and depression symptoms.

COVID-19-related worry was higher among students endorsing SCCs across any domain (*U* = 109458.5, *p* < 0.001), confidence in learning (*U* = 90785.5, *p* < 0.001), concentration (*U* = 115407.5, *p* < 0.001), muddled thoughts (*U* = 116383.5, *p* < 0.001), and memory recall (*U* = 106698.5, *p* < 0.001; see Figure 2). COVID-19-related worry did not significantly contribute to likelihood of endorsing overall SCCs after controlling for anxiety and depression. However, students with greater worry were more likely to endorse specific concerns about their confidence in learning (OR = 1.45, 95% CI = 1.02–2.05, *p* = 0.039) and memory recall (OR = 1.43, 95% CI = 1.02–2.01, *p* = 0.040), beyond the effects of anxiety and depression.

**Figure 3:**
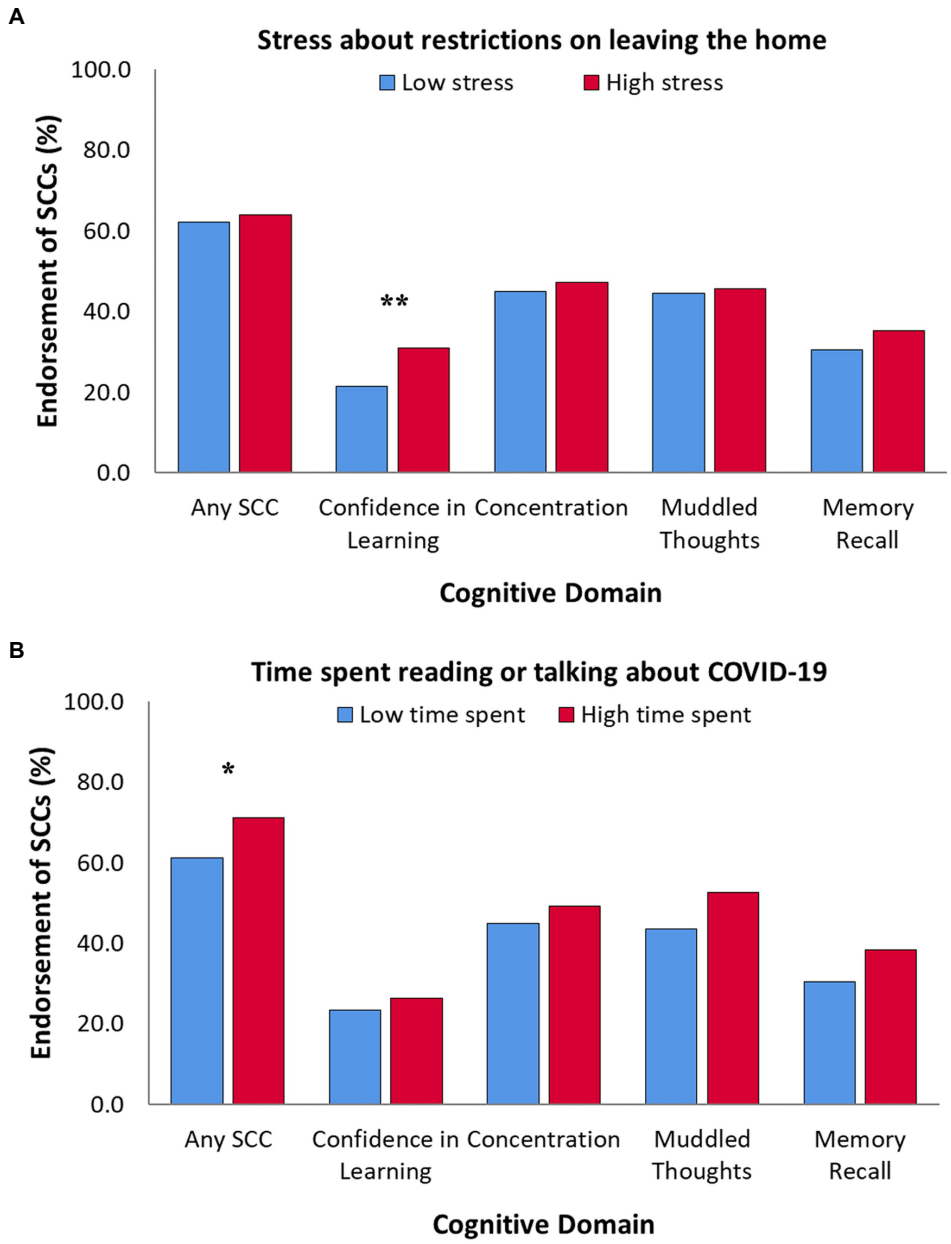
Percent endorsement of SCCs for each domain, between students **(A)** with low (*n* = 681) vs. high (*n* = 217) levels of reported stress about restrictions on leaving the home, and **(B)** spending low (*n* = 763) vs. high (*n* = 135) amounts of their time reading or talking about COVID-19. Percentages based on number of students with valid survey responses (i.e., excluding missing values), and not adjusted for differences in self-reported anxiety and depression symptoms; *p < 0.05, **p < 0.01.

Regarding stress about restrictions on leaving home, students did not differ on overall SCC endorsement. However, those reporting higher levels of stress endorsed specific concerns about confidence in learning (*p* = 0.006; Figure 3). Paradoxically, after controlling for anxiety and depression, students reporting higher stress were less likely to endorse overall SCCs (OR = 0.62, 95% CI = 0.43–0.90, *p* = 0.011), or specific concerns around concentration (OR = 0.67, 95% CI = 0.47–0.95, *p* = 0.023), muddled thoughts (OR = 0.58, 95% CI = 0.41–0.83, *p* = 0.003), and memory recall (OR = 0.65, 95% CI = 0.44–0.94, *p* = 0.022).

#### 3.4.2. Exposure prevention and hygiene anxiety

**Figure 4:**
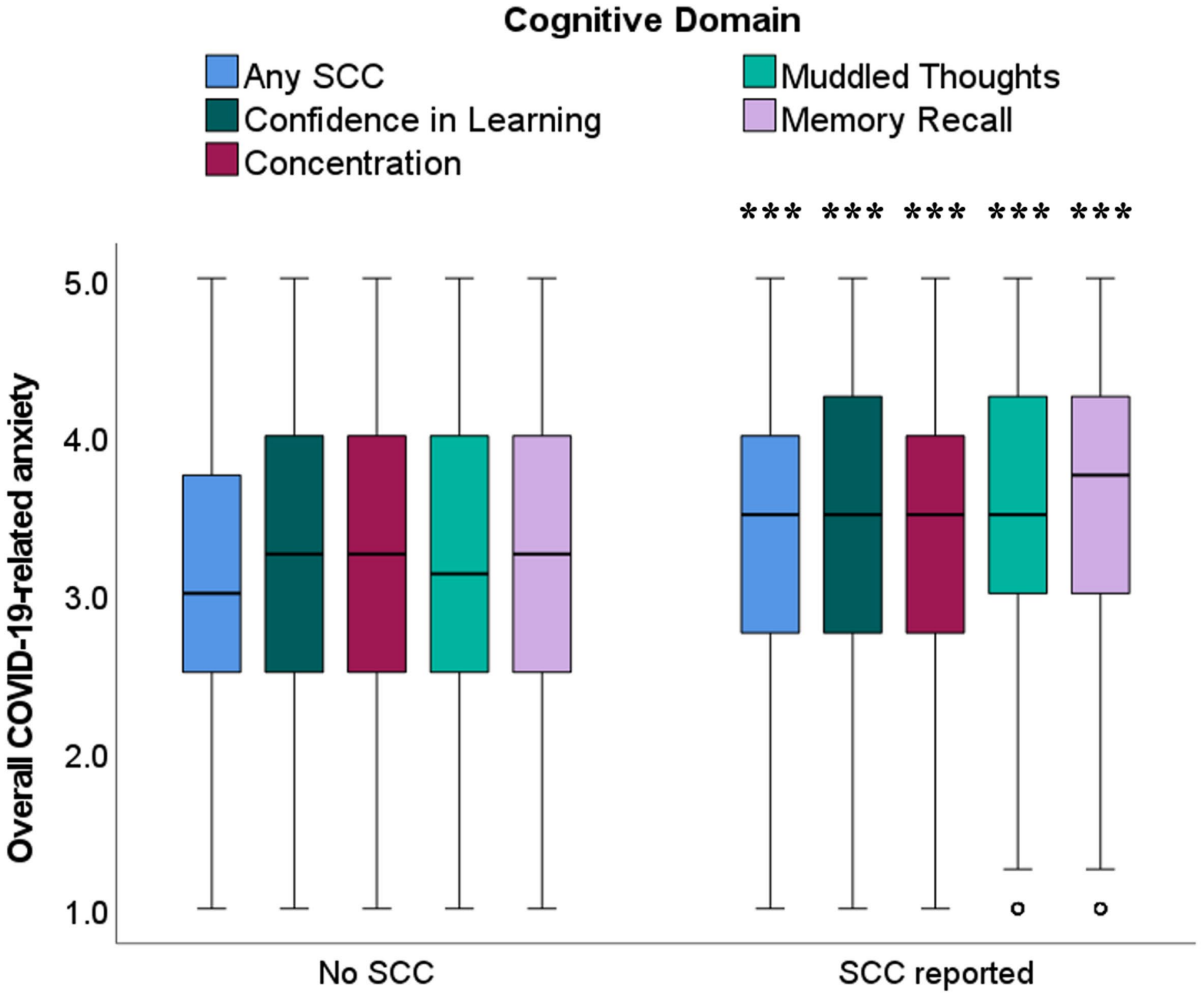
Boxplots illustrating higher median scores for anxiety related to COVID-19 exposure prevention and hygiene practices, in students reporting the presence of SCCs across all cognitive domains. ***p ≤ 0.001, for comparisons between students endorsing presence vs. absence of SCCs within each cognitive domain.

Level of anxiety about COVID-19-related exposure prevention and hygiene practices was higher among students endorsing SCCs in any domain (*U* = 110105.5, *p* < 0.001), confidence in learning (*U* = 83026.5, *p* = 0.001), concentration (*U* = 113299.0, *p* < 0.001), muddled thoughts (*U* = 118114.5, *p* < 0.001), and memory recall (*U* = 104490.5, *p* < 0.001; see Figure 4). Exposure prevention and hygiene anxiety did not significantly contribute to likelihood of endorsing overall SCCs after controlling for anxiety and depression.

#### 3.4.3. Time spent reading or talking about COVID-19

More time spent reading/talking about COVID-19 was associated with more frequent SCCs overall (*p* = 0.033; Figure 3). However, time spent did not significantly contribute to likelihood of endorsing overall SCCs after controlling for anxiety and depression.

### 3.5. Influence of university degree type and year level on SCCs

Students enrolled in an undergraduate degree were significantly younger (20.0 [19.0, 22.0] years) than postgraduate students (25.0 [24.0, 32.0] years; *U* = 28680.0, *p* < 0.001), and also more frequently endorsed SCCs overall (*p* = 0.005; Table 2).

**Figure 5:**
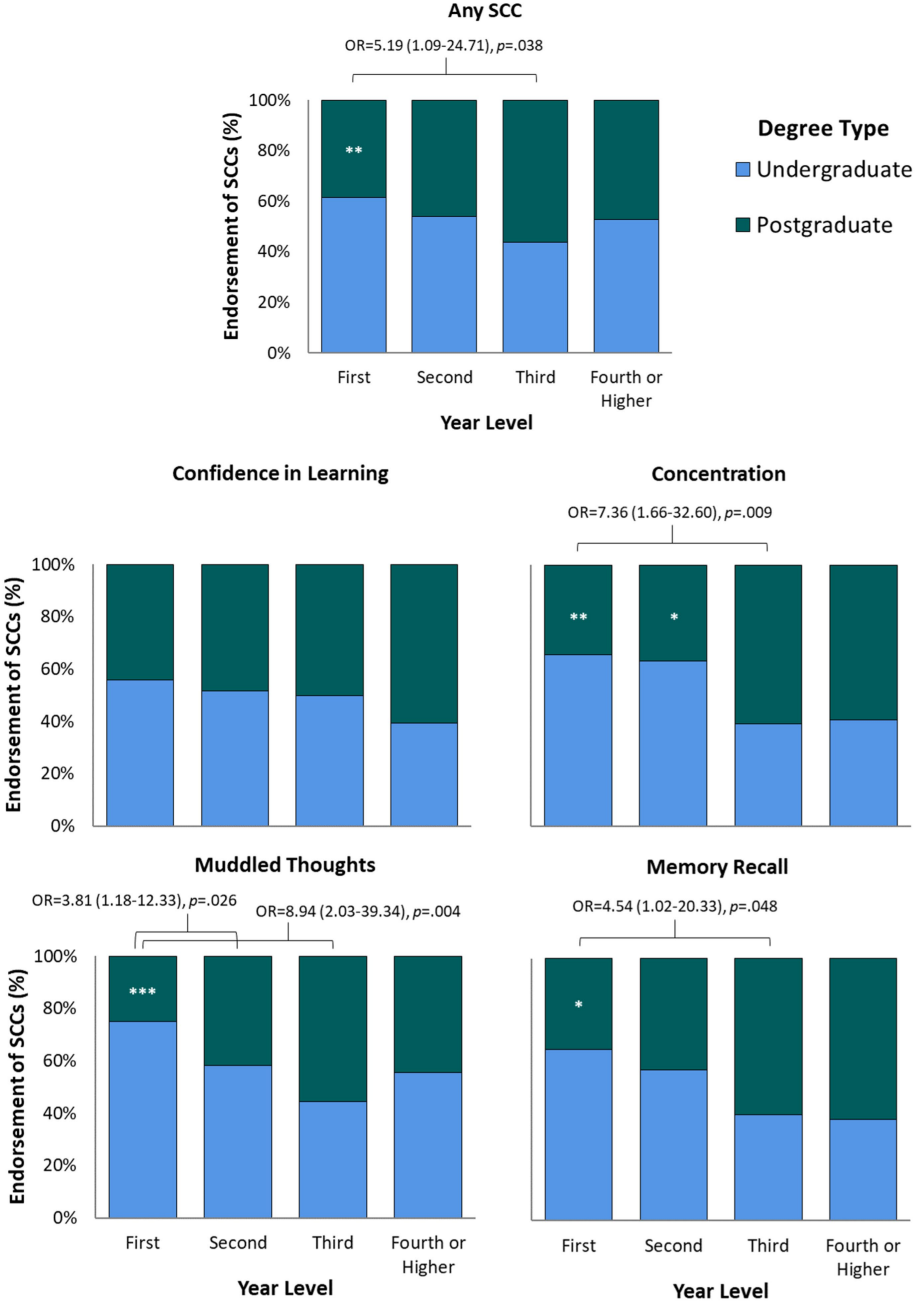
Endorsement of SCCs (scaled to 100%) across year level for undergraduate and postgraduate students. Based on number of students with valid survey responses (i.e., excluding missing values). Significant Year x Degree Type interaction for overall SCCs (71.4% first-year [Y1] undergraduates vs. 51.7% Y1 postgraduates), concentration (49.2% Y1 undergraduates vs. 25.8% Y1 postgraduates; and 50.8% second-year [Y2] undergraduates vs. 29.5% Y2 postgraduates), muddled thoughts (57.1% Y1 undergraduates vs. 19.1% Y1 postgraduates), and memory recall (36.5% Y1 undergraduates vs. 19.4% Y1 postgraduates), *p < 0.05, **p < 0.01, ***p < 0.001. Odds ratios (OR) with 95% confidence intervals displayed from hierarchical binary logistic regression analyses accounting for the influence of self-reported anxiety and depression scores on SCC endorsement (see main text).

Endorsement of SCCs was similar between students in the first, second, third, or fourth and higher year of their degree (Table 2). A significant interaction between degree type and year level was observed, such that SCCs were more frequently reported by undergraduate students in the earlier years of their degree, and by postgraduate students in the later years of study (Figure 5). This was statistically supported by a series of post-hoc Fisher’s exact tests comparing overall and domain-specific SCC endorsement between undergraduates and postgraduates at each year level. First-year undergraduates more frequently endorsed SCCs overall (*p* = 0.001), and specific concerns about concentration (*p* = 0.004), muddled thoughts (*p* < 0.001), and memory recall (*p* = 0.025), compared with first-year postgraduates (see Figure 5). Second-year undergraduates also frequently endorsed concerns about concentration (*p* = 0.031). Third-year and fourth-year undergraduate and postgraduate students did not significantly differ in their SCC endorsement for any cognitive domains.

Anxiety and depression scores significantly differed between students in different year levels. After controlling for anxiety and depression, SCC endorsement was more likely in first-year undergraduate students compared to third-year postgraduate students. This effect was observed for overall SCC endorsement, concentration, muddled thoughts, and memory recall (see Figure 5). Concerns about muddled thoughts were also more likely to be endorsed by first-year undergraduates compared to second-year postgraduates.

## 4. Discussion

This study aimed to characterize the nature of SCCs among Australian university students during the COVID-19 pandemic in December 2020, and examine demographic and COVID-19 correlates of SCCs. Over half the students surveyed perceived negative changes in cognition, with concerns about concentration and clarity of thoughts more prevalent than learning or memory, consistent with previous findings (Fiorenzato et al., 2021). Attention, concentration, and general thinking abilities were therefore vulnerable to disturbance in the context of intense or prolonged stress. It is possible that increased neuroinflammation in response to pandemic-related stressors may underlie cognitive complaints in non-infected individuals (Brusaferri et al., 2022), akin to the neurobiological responses associated with posttraumatic stress (Fourrier et al., 2019; Lee et al., 2022). The high prevalence of reported concentration difficulties is concordant with this being a prominent symptom of psychological disorders such as depression (Giusti et al., 2020), and thus may also reflect mental health concerns in students during the pandemic. However, perceived negative changes in cognition remained after controlling for depression and anxiety symptoms in the logistic regression models. This indicates that beyond emotional distress, students were experiencing distinct difficulties in cognitive functions. Despite their concerns, less than a quarter of students had spoken to someone about their cognition, emphasizing a critical need for monitoring, providing education to highlight and normalize links between cognitive and mental health difficulties, and increase support and strategies for university students in managing these challenges. Beyond supporting academic progress during COVID-19, improving awareness and management of cognitive concerns may have broader benefits for students within other stressful contexts (e.g., exam periods, natural disasters, or individual trauma experiences).

### 4.1. Demographic correlates

Our first hypothesis was partially supported. Even after accounting for anxiety and depression symptoms, we observed greater SCC endorsement in younger students, especially first-year undergraduates. This suggests vulnerability to experiencing cognitive difficulties, or less effective compensatory or coping strategies for managing perceived cognitive changes. Transition from school to tertiary education is associated with challenges including finding accommodation and affording food, independently managing new responsibilities, feeling homesick, and difficulties making new friends (Denovan and Macaskill, 2013; Knoesen and Naudé, 2018). First-year undergraduates in the present study had more than half of their 2020 academic year disrupted by pandemic restrictions and transition to remote learning. Undergraduates with at least one “normal” academic year under their belt may have felt more confident in their existing learning abilities and study habits, or have more established university-based social support networks. Conversely, the impact of pandemic-induced restrictions on face-to-face human research, reduced access to laboratory spaces and equipment, and postponement of clinical and/or industry placements, may have been more profound for postgraduate students in the later years of their programs. Postgraduate students near completion may experience different demands on their time or additional pressures (e.g., caregiving, financial) that contribute to a greater cognitive burden. Ongoing assessment of changes in students’ mental and cognitive wellbeing will be essential to more accurately gage how SCCs and psychological distress are related to academic challenges, and how SCCs fluctuate with periods of easing or tightening of COVID-19 restrictions.

There was no systematic increase in SCCs among female students (despite the higher proportion of female respondents), contrasting recent studies (Fiorenzato et al., 2021; Giusti et al., 2021). Giusti et al. (2020) found no gender-based differences in endorsement of concentration issues assessed *via* the relevant items of two measures of psychological distress and depression. Similarly, Podlesek et al. (2021) found no difference in cognitive change scores between females and males, although females endorsed greater stress responses (negative emotions, anxiety, perceived stress, physical symptoms), which subsequently predicted SCC. Future investigations in university students should examine cognitive, emotional, and physical health side-by-side, to verify mediating factors that may identify students at most risk of cognitive difficulties. Further exploration might also uncover gender differences in students’ perceptions of stigma and willingness to disclose mental health versus cognitive health concerns (e.g., Brown et al., 2018).

Greater SCCs were associated with White/European ethnicity, corroborating the increased risk for psychological wellbeing in these students (Liu et al., 2021). Differences in emotion-related thinking styles has been associated with different inclinations toward Western versus Asian cultural values in undergraduate students (Dere et al., 2012), and may have contributed to students’ understanding and reporting of mental and cognitive health issues (Liu et al., 2021). Mental health literacy (knowledge and beliefs about mental illness) is variable across non-Western countries and cultures (Furnham and Hamid, 2014; Furnham and Swami, 2018), and individuals with lower mental health literacy are likely less able to recognize subtle changes in day-to-day cognitive processing. Although our survey items assessing perceived changes in cognition were worded to minimize technical terminology or jargon, further exploration may illuminate how students’ understanding varies as a function of ethnic background or cultural values, and the subsequent influence on rates of reported SCCs.

More broadly, an optimistic thinking style or resilient mindset has been associated with lower psychological distress and endorsement of cognitive concerns among students during the pandemic (Giusti et al., 2020; Arslan and Coşkun, 2022). Validation of emotional, cognitive, and personality-related factors moderating students’ stress responses to the pandemic situation will prove valuable for the development of age- and culturally-appropriate, effective, and efficient prevention and intervention programs that can be implemented on a large, university-wide scale.

### 4.2. COVID-19-related correlates

Concerns about learning and memory were more frequent among students with greater worry about various impacts of COVID-19, even after controlling for anxiety and depression symptoms. This accords with previous studies showing that lockdown confinement and COVID-19-related worry are related to increased anxiety, depression, and stress (Blix et al., 2021; Podlesek et al., 2021; Wilson et al., 2021), and academic difficulties in students with high COVID-19-related anxiety (Giusti et al., 2021). No additional effects of exposure prevention and hygiene-related anxiety on SCC endorsement were observed, nor were any relationships between reading or talking about COVID-19 and SCCs. The impact of COVID-specific distress therefore appears closely related to general psychological distress, further highlighting those students with higher self-reported symptoms as an at-risk group.

Taken together, these results indicate a high prevalence of cognitive concerns across vulnerable student subgroups, with the majority of these students reporting they did not seek advice about their concerns. An important consideration in interpreting these results is that students completed this survey during university summer holidays, several weeks following Victoria’s longest and harshest lockdown. High rates of SCCs reflect a possible cumulative burden of COVID-19 restrictions on subjective cognitive difficulties in university students. Worry about grades, future academic success and job opportunities may not be immediately resolved with the end of lockdown. Upon Victoria’s return to relative “COVID normality,” high case numbers in other countries and continuing (negative) media coverage of the pandemic may have served to prolong stress and worry in students and the broader community. Restrictions on travel remained in place at the end of 2020, limiting opportunities to visit families and recuperate from the effects of lockdown. In addition, remote online study activities contributed to burnout and fatigue (e.g., “Zoom fatigue”; Nesher Shoshan and Wehrt, 2021; Peper et al., 2021), which can impact academic performance (Schaufeli et al., 2002).

### 4.3. Strengths and limitations

This is the first study investigating the cognitive impact of COVID-19 in Australian university students, with a large sample allowing us to explore SCCs across multiple demographic and university subgroups. Given the positive relationships between psychopathological and cognitive symptoms (Monastero et al., 2009; Podlesek et al., 2021; Perin et al., 2022), this study demonstrated that SCCs are still highly prevalent in specific at-risk cohorts (younger, first-year undergraduates, White/European ethnic backgrounds). Although two years on, lockdown restrictions in many countries have now eased, the 2020 data presented here remain valid and important for a couple of key reasons. Current COVID-19 control measures are still variable and fluctuating worldwide (e.g., comparing the COVID-19 Stringency Index between Australia, United States, and China at the end of 2022; see Mathieu et al., 2020; Hale et al., 2021). In the absence of substantial extant literature on the cognitive impacts of a pandemic, it is plausible that these effects are ongoing within more vulnerable subgroups, and may reoccur with future exacerbations of the COVID-19 situation specifically, or other as yet unpredictable pandemic, epidemic, or natural disaster events. Consequently, the cognitive impact of COVID-19 restrictions in 2020 provide a baseline from which to further assess the severity and longevity of students’ cognitive concerns. Self-reported changes in everyday cognitive abilities may represent an additional potential marker of underlying mental health issues or distress. This should encourage more effective assessment, monitoring, and intervention for cognitive concerns, both within the ongoing COVID-19 situation, and in the context of future community- or nation-wide major stressors or disasters.

As lockdown restrictions prompted a halt in non-essential face-to-face human research, it was not feasible to administer tests of objective cognitive functioning, nor was ethical approval obtained to access students’ academic records. Thus, this study is limited by its use of subjective measures of cognitive ability. It also remains unclear to what extent students’ SCCs reflected academic performance outcomes. Poorer objective and subjective cognition have previously been reported among undergraduate students during the pandemic, and subjective and objective indicators of cognition are highly related (Crumley et al., 2014; Burmester et al., 2016). However, such associations are often moderated by depression symptoms and other demographic and measurement-related factors (Crumley et al., 2014; Srisurapanont et al., 2017).

The interpretation of our study findings is limited to a tertiary education sample comprising predominantly female students. Although a higher proportion of females is consistent with other recent investigations of students (Giusti et al., 2020, 2021; Wang et al., 2020; Arslan and Coşkun, 2022) and general community populations (Rossi et al., 2020; Fiorenzato et al., 2021; Podlesek et al., 2021) during COVID-19, future research warrants a more thorough examination of the unique pandemic-related mental and cognitive health issues faced by males, and specifically male university students.

### 4.4. Conclusion

COVID-19 necessitated a multitude of adaptations to traditional methods of teaching and learning across universities worldwide. By capturing the potentially cumulative effects of COVID-19 restrictions on perceptions of cognitive function in university students, this study highlighted a concerning proportion of students perceiving negative cognitive changes several weeks post-lockdown. Higher education institutions need to support student wellbeing and academic goals through monitoring of cognitive concerns particularly in high-risk subgroups, provision of appropriate resources and services for identifying and managing mental, cognitive, and physical health concerns, and clear communication of relevant restrictions and their impacts on academic activities. Mental health awareness campaigns are prevalent across many modern Western societies. Awareness around recognizing and managing cognitive health concerns is comparatively lacking, and implicates a need for further promotion, particularly in vulnerable subgroups such as students. Pandemic control measures such as physical distancing and social isolation have only further emphasized the utility of digital technology-based tools and interventions for health and wellbeing among the general population, in addition to specific cohorts such as university students (Harith et al., 2022), individuals with dementia (Bird and Lim, 2022), and rural, regional, and remote communities (O’Kane, 2020). Such resources (e.g., audio-visual telehealth platforms, smartphone applications, web-based forums or groups) are useful in connecting individuals to information, self-help strategies, health professionals, or peers, during periods of lockdown or restricted access. Beyond the pandemic context, digital technology can generally extend the reach of clinical and research-based resources and services, making it an ideal method for delivering psychological interventions to improve mental and cognitive health in young, vulnerable student subgroups, at institution-wide and nation-wide levels.

The ongoing THRIVE@Monash survey series will explore the longer-term effects of fluctuating COVID-19 restrictions on students’ cognitive health, as further lockdowns and disease control measures were implemented throughout 2021 and 2022.

## Data availability statement

The raw data supporting the conclusions of this article will be made available by the authors upon reasonable request. The data are not publicly available due to privacy.

## Ethics statement

The studies involving human participants were reviewed and approved by Monash University Human Research Ethics Committee. The patients/participants provided their written informed consent to participate in this study.

## Author contributions

MM and KC: conceptualization. MM, YL, and KC: methodology. LB: formal analysis, writing—original draft, and visualization. MM: investigation, data curation, and project administration. LB, MM, YL, and KC: writing—review and editing. KC: supervision. All authors contributed to the article and approved the submitted version.

## Conflict of interest

The authors declare that the research was conducted in the absence of any commercial or financial relationships that could be construed as a potential conflict of interest.

## Publisher’s note

All claims expressed in this article are solely those of the authors and do not necessarily represent those of their affiliated organizations, or those of the publisher, the editors and the reviewers. Any product that may be evaluated in this article, or claim that may be made by its manufacturer, is not guaranteed or endorsed by the publisher.
