# Supplementary material for: Prevalence and correlates of subjective cognitive concerns in Australian university students during the COVID-19 pandemic
Source: Front Psychol. 2023 Jan 11;13:1094497. doi: 10.3389/fpsyg.2022.1094497 (PMC9874933; doi:10.3389/fpsyg.2022.1094497)
Supplement: Supplementary file 1 [file Data_Sheet_1.docx]

Supplementary Material

# Methods

## Measures

### Subjective cognitive concerns

Participants were asked to reflect on perceived cognitive changes in relation to the teaching semester prior to the THRIVE@Monash survey launch.

Four items presented participants with a 5-point Likert scale to rate the extent of perceived changes in domains of “confidence in your ability to learn new skills or complex programs” (referred to as “confidence in learning”), “ability to concentrate for sustained periods of time” (concentration), “thoughts becoming more muddled” (muddled thoughts), and “ability to recall important information” (memory recall). Scale responses ranged from 1 = *much less confident* or *much worse/more* to 5 = *much more confident* or *much better/less*, with 3 = *about the same* indicating no changes. Binary scores in each domain were derived by collapsing responses indicating worsened ability (presence of SCC) and responses indicating no change or better ability (no SCC). A similar binary score collapsed across all 4 domains reflected global presence or absence of SCCs (i.e., whether a participant endorsed any cognitive concern on any of the 4 items).

In addition, two ‘yes/no’ questions asked participants in general if they had “any concerns about your thinking and memory”, and consequently for a response of ‘yes’, “have you spoken with a health professional about these concerns?”.

### COVID-19 exposure and worry

Participants were asked if anyone within or outside of their household had been diagnosed with COVID-19. To note, at the time of the survey there was no active COVID-19 transmission in the community in most of Australia.

One set of questions asked participants to rate their level of worry (1 = *never* to 5 = *extremely*) regarding 11 COVID-19-related concerns: infection of self or friends and family, impact of COVID-19 on physical or mental health, having enough money and resources, staying safe when leaving the house, how Australia or the world will look after the pandemic, and whether self or others were doing enough to slow the spread of COVID-19 at the time. As scores on these 11 items were all significantly positively correlated, a mean score was calculated to reflect overall level of COVID-19-related worry.

One set of questions explored anxieties relating to COVID-19 exposure prevention and hygiene practices. Participants responded 1 = *strongly disagree* to 5 = *strongly agree* for 4 statements about anxiety when “I am in a large group of people”, “I see someone not wearing a face mask in public”, “someone coughs or sneezes near me”, or “I cough or sneeze”. As these items were all significantly positively correlated, responses were again averaged into a composite score reflecting overall anxiety related to these COVID-19 exposure prevention and hygiene practices.

Two questions assessed self-reported time spent reading or talking about COVID-19 (1 = *no time* to 5 = *most of my time*), and level of stress regarding restrictions on leaving the home (1 = *not at all* to 5 = *extremely*). The distribution of scores on both of these variables was significantly positively skewed, with minimal change across a number of statistical transformations. For each variable we therefore used a median split to compare students reporting low versus high levels of time spent reading/talking about COVID (median response=3, *some time*), or stress regarding restrictions (median response=2, *slightly stressed*).

### Anxiety and depression

The Patient-Reported Outcomes Measurement Information System (PROMIS) Anxiety (8a) scale focuses on feeling nervous, fearful, tense, and overwhelmed. The PROMIS Depression scale focuses on feelings of depression, helplessness, worthlessness and hopelessness. PROMIS Anxiety and Depression scales have been widely validated and tested in both clinical (Schalet et al., 2016) and broader populations (Choi et al., 2014; Schalet et al., 2014). Responses regarding the frequency of experiences in the past 7 days are scored on a 5-point Likert scale (1 = *never* to 5 = *always*), producing total raw scores ranging from 8-40, and converting to T-scores ranging from 37.1-83.1 for Anxiety and 38.2-81.3 for Depression. The PROMIS Anxiety and PROMIS Depression (short form) showed strong internal validity with a Cronbach’s alpha of .89 and .93, respectively (Kroenke et al., 2014). In the present study, Cronbach’s alpha was .96 for both the PROMIS Anxiety and Depression scales.

# References

Schalet, B. D., Pilkonis, P. A., Yu, L., Dodds, N., Johnston, K. L., Yount, S., Riley, W., Cella, D. (2016). Clinical validity of PROMIS Depression, Anxiety, and Anger across diverse clinical samples. *Journal of Clinical Epidemiology, 73*, 119-127. https://doi.org/10.1016/j.jclinepi.2015.08.036

Choi, S. W., Schalet, B., Cook, K. F., & Cella, D. (2014). Establishing a common metric for depressive symptoms: Linking the BDI-II, CES-D, and PHQ-9 to PROMIS Depression. *Psychological Assessment, 26*(2), 513-527. https://doi.org/10.1037/a0035768

Schalet, B. D., Cook, K. F., Choi, S. W., & Cella, D. (2014). Establishing a common metric for self-reported anxiety: Linking the MASQ, PANAS, and GAD-7 to PROMIS Anxiety. *Journal of Anxiety Disorders, 28*(1), 88-96. https://doi.org/10.1016/j.janxdis.2013.11.006

Kroenke, K., Yu, Z., Wu, J., Kean, J., & Monahan, P. O. (2014). Operating characteristics of PROMIS four-item Depression and Anxiety scales in primary care patients with chronic pain. *Pain Medicine, 15*(11), 1892-1901. https://doi.org/10.1111/pme.12537

# Supplementary Figures

#
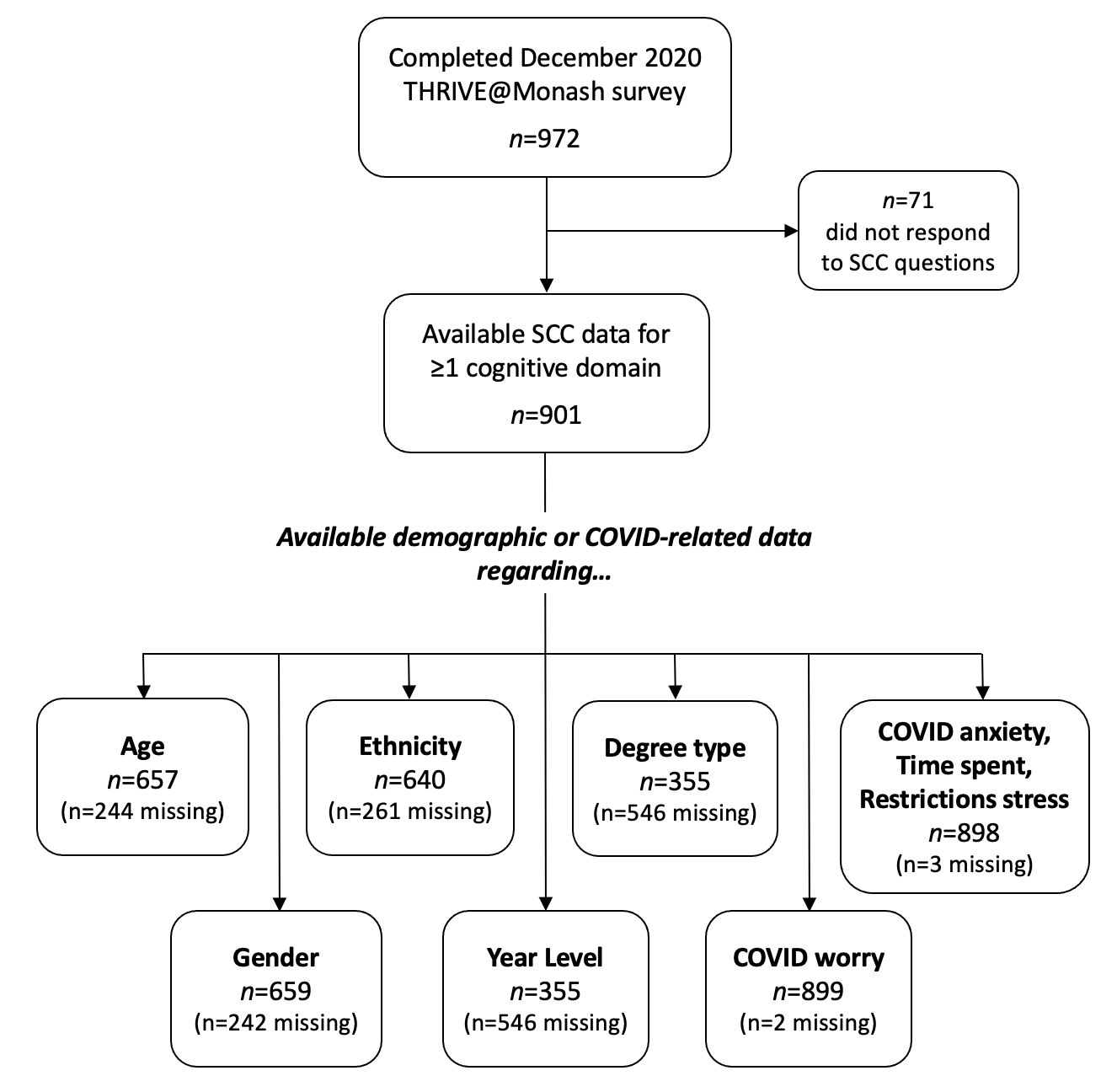


**Supplementary Fig. 1.** Participant flowchart illustrating missing data across subjective cognitive concerns (SCCs), demographic, enrolment, and COVID-19-related variables of interest. Time spent=time spent reading or talking about COVID-19; Restrictions stress=stress regarding restrictions on leaving the home.
